# The significance of routine biochemical markers in patients with major depressive disorder

**DOI:** 10.1038/srep34402

**Published:** 2016-09-29

**Authors:** You-Fan Peng, Yang Xiang, Ye-Sheng Wei

**Affiliations:** 1 Department of Laboratory Medicine, Affiliated Hospital of Youjiang Medical University for Nationalities, No. 18 Zhongshan Er Road, Baise, Guangxi 533000, China

## Abstract

The aim of our study is to examine the levels of routine biochemical markers in patients with major depressive disorder (MDD), and combine multiple biochemical parameters to assess the discriminative power for patients with MDD. We used the Hamilton Depression (HAMD) score to evaluate the severity of depressive symptoms in 228 patients with MDD. The phase of depression severity was between moderate and severe in MDD patients. There were significant differences between MDD patients and healthy controls in alanine transaminase (ALT), urea nitrogen (UN), lactate dehydrogenase (LDH), uric acid (UA), total protein (TP), total bile acid (TBA), creatinine (Cr), total bilirubin (Tbil), direct bilirubin (Dbil) and indirect bilirubin (Ibil), high density lipoprotein-cholesterol (HDL-C), fasting blood-glucose (FBG) and fructosamine (SF). Multivariate analysis showed that UN, FBG, HDL-C, SF, TP, Cr and Tbil remained independently association with MDD. Further, a logit equation was established to identify patients with MDD. The composite markers exhibited an area under the curve of 0.810 with cut-off values of 0.410. Our results suggest the associations between UN, FBG, HDL-C, TP, Cr, Tbil, SF and MDD, use of these routine biochemical markers in combination may contribute to improve the complete management for patients with MDD.

Depression is a mental disease with global public health concern, especially in developing countries^1^. There was evidence that up to 6–12% of the adult population suffered mental disorder and recurrent depression^2^. Notable, the etiological research of depression has aroused widespread interest in recent years. Among some studies, a plausible association between depressive disorder and glial cell line-derived neurotrophic factor has been well-established by Michel TM^3^. In addition, researchers have speculated that dysregulated immune response system may be involved with the pathogenesis of major depressive disorder (MDD)^4^. In another study, oxidative stress has been considered as the physiopathologic mechanism of depressive disorder^5^. A recent cross-sectional study found that depression was related to chronic inflammation characterized by elevated C-reactive protein (CRP), interleukin-6 (IL-6) and tumor necrosis factor (TNF)^6^. It has been previously observed that use of TNF inhibitor can alleviate depressive symptoms for treatment-resistant MDD patients with elevated inflammatory markers^7^, and the anti-inflammatory medications such as cyclooxygenase-2 inhibitor celecoxib exhibited a satisfactory therapeutic effect in patients with MDD^8^.

Recently, to diagnose neuropsychiatric disorders such as depressive disorder, schizophrenia and bipolar disorder, proteomic technology has been developed as a useful tool to identify MDD patients^9^. Secondly, near-infrared spectroscopy has also been regarded to be a reliably laboratory test for the diagnosis of MDD^10^. However, these tools are uncommon and unavailable in the clinical laboratory. Recent studies have shown that several laboratory markers are associated with systemic inflammation and oxidative stress, such as urea nitrogen (UN), creatinine (Cr), fructosamine (SF) and bilirubin^11^,^12^. Unfortunately, their single use is often limited by poor sensitivity and specificity. Therefore, the aim of our study is to investigate the levels of routine biochemical markers in MDD patients, and combine multiple biochemical biomarkers to estimate the discriminative power for patients with MDD.

## Materials and Methods

Laboratory and demographic data were analyzed in 228 patients with MDD, wherein included 43 male and 185 female. The diagnosis of MDD was determined by DSM-IV criteria^13^,^14^. All patients accompanied by cardiovascular disease, hypertension, endocrine disease, liver and kidney disorder, gout, infectious disease, metabolic syndrome, autoimmune disease, malignancy, pregnancy and head trauma history were not included, and any patients with anxiety disorders, neurodegenerative disorders, bipolar or psychotic disorders, mental retardation, psychiatric medications use and substance abuse were also excluded. We used the Hamilton Depression (HAMD) score to evaluate the severity of depressive symptoms^15^, higher scores on the HAMD expressed more severe symptomatology. The following severity range for the HAMD score was used to classify the depressive symptom severity: mild depression (8–16); moderate depression (17–23); and severe depression (≥24)^16^.

A total of 251 healthy subjects with healthy diet at least one month were selected as controls, and all healthy individuals were no history of head trauma history, psychiatric disorder, neurological disorder in this study. The study was performed in accordance with the Declaration of Helsinki, and approved by the Ethics Committee of the Affiliated Hospital of Youjiang Medical University for Nationalities, and informed consent was obtained from all individuals.

Demographic and clinical data were obtained from diagnostic records. The body mass index was calculated as an individual’s weight in kilograms divided by the square of height in meters. Fasting venous blood from participants were collected to measure biochemical parameters, including alkaline phosphatase (ALP), total cholesterol (TC), alanine transaminase (ALT), aspertate aminotransferase (AST), UN, low density lipoprotein-cholesterol (LDL-C), high density lipoprotein-cholesterol (HDL-C), γ-glutamyl transpeptidase (γ-GGT), glucose, lactate dehydrogenase (LDH), uric acid (UA), SF, direct bilirubin (Dbil), total bile acid (TBA), total bilirubin (Tbil), total protein (TP), triglyceride (TG), indirect bilirubin (Ibil) and Cr. All biochemical tests were performed by automatic biochemical analyzer.

### Statistical analysis

Statistical analyses were performed with the statistical package SPSS version 16.0. All continuous variables were expressed as mean (±standard deviation), and categorical variables were expressed as percentage. A significant calculation for sample size was performed by Quanto software. The normality of data was tested with Kolmogorov-Smirnov test. The differences between continuous variables were compared by student t test or Mann-Whitney U test as appropriate. Demographic data were compared by Chi-square test. We used stepwise logistic regression analysis to identify underlying biochemical parameters associated with depressive disorder. Hosmer-Lemeshow test was also used to examine an identified effectiveness of model. An identified performance of the combinations of biochemical markers was analyzed by receiver operating characteristic (ROC) curve. P < 0.05 was considered statistically significant.

## Results

All patients were from Chinese Hans population in the identical regions, the mean age of patients with MDD was 39.6 years and 185 (81.1%) were females. Regarding the cumulative clinical profile, the phase of depression severity was between moderate (68.9%) and severe (31.1%) in accordance with HAMD total score (21.7 ± 4.12), almost patients suffered from somatic and psychiatric comorbidities, such as pain (56.1%), poor appetite (63.2%), fatigue (37.7%), palpitations (50.0%), insomnia (65.4%), hallucination (42.1%), delusion (37.3%), guilt (26.8%) and psychomotor retardation (24.6%). There were no statistically significant differences in gender, age and body mass index between MDD patients and controls, as shown in Table 1.

Cumulative results for biochemical tests were obtained from patients with MDD and controls. The laboratory characteristics were outlined in Table 2. Several significant differences were observed between the two groups, lower values of ALT, UN, LDH, UA, TP, TBA, Cr, Tbil, Dbil, Ibil were found to be statistical significance in patients with MDD compared with controls. In contrast, the levels of HDL-C, fasting blood-glucose (FBG) and SF were higher in patient with MDD than controls. The other laboratory markers had not significant differences between the two groups.

Statistically significant variables in univariate analysis were considered into stepwise logistic regression analysis, the results of logistic regression analysis showed that UN, FBG, HDL-C, SF, TP, Cr and Tbil remained independently association with MDD (Table 3). Regression coefficients of these biochemical markers were used to calculate a logit equation for the assessment of patients with MDD as follows: The logarithm of odds = 0.511 − 0.294 (UN) + 1.537 (FBG) + 1.009 (HDL-C) + 2.379 (SF) − 0.139(TP) − 0.108 (Tbil) − 0.043 (Cr).

This calculated model was evaluated by using Hosmer-Lemeshow test (P = 0.325, Chi-square = 9.212). To evaluate the performance of combined biomarkers for patients with MDD, the sensitivity, specificity and area under the curve (AUC) of these markers in combination were calculated, respectively (Table 4). The composite markers exhibited an AUC of 0.810 (95% CI: 0.796–0.846, P < 0.001) with the sensitivity of 0.806 and specificity of 0.636, and a cut-off values was defined with 0.410, indicating a more better effectiveness in identifying patients with MDD than all single markers.

## Discussion

To this day, there are few objective and available laboratory markers to estimate completely pathological conditions in patients with MDD, and single laboratory marker is often the lack of well sensitivity and specificity. Clinical biochemical tests are routine hospital examinations in clinical practice. This investigation found that UN, FBG, HDL-C, SF, TP, Cr and Tbil were independently associated with MDD in logistic regression analysis. Further, our study revealed that use of these markers in combination could provide useful and objective information in the assessment for patients with MDD.

Oxidative stress derives from increased production of reactive oxygen species, which leads to cell damage by biological reactions such as lipid peroxidation, enzyme inactivation and DNA modification^17^. Previous studies have demonstrated that oxidative stress related enzymes are associated with the pathogenic process of patients with depression, and anti-oxidative enzymes activity is increased in patients suffering from depressive disorder^18^,^19^. Emerging evidence has suggested that several inflammatory cytokines such as interleukin-1 (IL-1) beta, IL-6 and TNF are implicated with oxidative stress in patients with MDD^20^,^21^. Interesting, oxidative stress process has also been observed in the frontal cortex of patients with recurrent depressive disorder^22^. Moreover, the xanthine oxidase activity is increased in the thalamus and the putamen of patients with depression, which tends to induce oxidative stress by an increased production of reactive oxygen species^23^. These reports provide an important fact that oxidative stress may be a major contributor in the pathogenesis of MDD. There is no literature available with respect to serum bilirubin levels in patients with MDD, serum bilirubin levels in patients MDD were found to be decreased, and lower Tbil concentrations were associated with MDD in the present study. Bilirubin, the end product of heme catabolism, is an efficient and powerful antioxidant, the role of bilirubin in regarding with oxidation resistance, anti-inflammation and immunosuppression has been demonstrated in various diseases^24^. Indeed, lower serum levels of bilirubin have been reported in patients with migraine, carbon monoxide-poisoned and pulmonary embolism^25^,^26^,^27^,^28^, and serum bilirubin may provide a protective action in patients with cardiovascular disease and rheumatic disease^29^,^30^. In these studies, bilirubin as an endogenous antioxidant may be destroyed by excessive oxidative stress. Thus, the presence of oxidative stress may result in overconsumption of serum bilirubin, which is associated with lower bilirubin in patients with MDD.

Higher levels of HDL-C have been reported in patients with depression^31^. In agreement with this finding, increased levels of HDL-C were demonstrated to be associated with MDD patients in our study. In addition, we observed lower concentrations of UN and TP in patients compared to healthy controls, the findings are consistent with recently reports on MDD patients^32^. Serum Cr concentrations were decreased in MDD patients compared with controls, the results may attribute to poor appetite and nutrition in patients with MDD, because accompanied anorexia has a high prevalent in patients with MDD^33^,^34^. Of note, increased levels of FBG and SF were found to be associated with MDD in our study. Oxidative stress is a crucial player in the establishment of insulin resistance and diabetes mellitus^35^. In fact, oxidative stress has been regarded as an underlying mediator of insulin resistance, and there is an inversely relation between oxidative stress and insulin action^36^,^37^. Studies have shown that oxidative stress can decrease insulin sensitivity by GLUT-4 deficiency^38^. Furthermore, major insulin secretion has been found to be dependent on the regulation of intracellular and extracellular reactive oxygen species to a certain extent^39^. It has recently been shown that oxidative stress may increase induction of HO-1 expression, leading to insulin resistance and insufficient insulin^35^. Obviously, enhanced oxidative stress may result in insulin resistance and influence on insulin secretion in patients with depressive disorder. Nevertheless, elevated FBG and SF have been positively correlated with insulin resistance and insufficient insulin response in diabetic and non-diabetic patients^40^, which are associated with increase in serum FBG and SF concentrations in patients with MDD.

Our study provides an insight on the role of combination markers of UN, FBG, HDL-C, SF, TP, Cr and Tbil in patients with MDD. Specific and sensitive laboratory indexes have been limited in the diagnosis of depressive disorder. In clinical practice, the diagnosis of MDD main depends on physician’s clinical experiences, clinical symptoms and patient’s self-assessments. In the current study, these biochemical parameters are objective and available, the composite information from these routine biochemical markers may improve the diagnostic effectiveness of depressive disorder.

We are aware that this study has several limitations. First, our study only included Chinese Han nationality in this cross-sectional design. Second, the differences of dietary habit might have effect on biochemical parameters in different regions and groups, which limit the extrapolation effect of our results. Third, the diagnostic power had to be improved for the logarithm of odds, because other clinical information was not obtained as variables in multivariate analysis. Taken together, our results suggest the associations between UN, FBG, HDL-C, SF, TP, Cr, Tbil and MDD, use of these biochemical markers in combination may contribute to improve the complete management for patients with MDD. However, our results are needed to be further established with multicenter and larger-scale study.

## Additional Information

**How to cite this article**: Peng, Y.-F. *et al*. The significance of routine biochemical markers in patients with major depressive disorder. *Sci. Rep.* **6**, 34402; doi: 10.1038/srep34402 (2016).

## Figures and Tables

**Table 1:** Demographic and clinical characteristics of patients with major depressive disorder and controls.

	MDD patients	Healthy controls	P -value
	N = 228	N = 251	
Female, n (%)	185 (81.1)	201 (80.1)	0.769
Age (yr)	39.55 ± 8.06	39.97 ± 7.37	0.558
Body mass index (kg/m^2^)	24.34 ± 2.86	24.70 ± 3.01	0.185
Work, n (%)			
Full-time	86 (37.7)	—	—
Farmer	40 (17.5)	—	—
Student	13 (5.7)	—	—
Retired	12.7 (29)	—	—
Unemployed	60 (26.3)	—	—
Comorbidities s, n (%)			
Pain symptoms	128 (56.1)	—	—
Poor appetite	144 (63.2)	—	—
Fatigue	86 (37.7)	—	—
Palpitations	114 (50.0)	—	—
Insomnia	149 (65.4)	—	—
Hallucination	96 (42.1)	—	—
Delusion	85 (37.3)	—	—
Guilt	61 (26.8)	—	—
Psychomotor retardation	56 (24.6)	—	—
HAMD score	21.7 ± 4.12	—	—

**Table 2:** Biochemical parameters between MDD patients and controls in clinical laboratory.

	MDD patients	Controls	P-value
High density lipoprotein-cholesterol (mmol/L)	1.31 ± 0.32	1.24 ± 0.300	0.008
Low density lipoprotein-cholesterol (mmol/L)	2.53 ± 0.59	2.53 ± 0.68	0.957
Total cholesterol (mmol/L)	4.14 ± 0.70	4.08 ± 0.81	0.320
Triglyceride (mmol/L)	1.00 ± 0.35	0.97 ± 0.33	0.383
Alkaline phosphatase (U/L)	64.34 ± 18.67	66.77 ± 12.26	0.140
Alanine transaminase (U/L)	18.13 ± 11.59	21.17 ± 13.30	0.008
Aspertate aminotransferase (U/L)	17.40 ± 6.38	18.28 ± 6.05	0.122
γ-glutamyl transpeptidase(U/L)	19.88 ± 19.94	21.44 ± 16.37	0.348
Fasting blood-glucose (mmol/L )	4.73 ± 0.45	4.52 ± 0.43	<0.001
Fructosamine (mmol/L )	2.28 ± 0.27	2.19 ± 0.29	<0.001
Urea nitrogen (mmol/L )	4.33 ± 1.36	4.85 ± 1.23	<0.001
Lactate dehydrogenase (U/L)	147.94 ± 23.65	152.29 ± 23.75	0.046
Uric acid (μmol/L)	256.70 ± 69.84	285.22 ± 77.17	<0.001
Total protein (g/L)	66.72 ± 5.10	68.72 ± 5.23	<0.001
Creatinine (μmol/L)	56.62 ± 11.82	65.19 ± 14.09	<0.001
Total bilirubin (μmol/L)	8.31 ± 3.17	10.07 ± 4.50	<0.001
Direct bilirubin (μmol/L)	3.41 ± 1.20	3.72 ± 1.53	0.016
Indirect bilirubin (μmol/L)	4.90 ± 2.56	6.36 ± 3.73	<0.001
Total bile acid (μmol/L)	3.20 ± 2.57	4.49 ± 4.01	<0.001

**Table 3:** Variables associated with MDD patients in logistic regression analysis.

Biomarker	B	SE	Wald	P-value	OR	95% CI
Urea nitrogen (mmol/L )	−0.294	0.092	10.298	0.001	0.745	0.623–0.892
Fasting blood-glucose (mmol/L )	1.537	0.264	33.762	<0.001	4.649	2.769–7.807
Fructosamine (mmol/L )	2.379	0.450	27.982	<0.001	10.799	4.472–26.076
High density lipoprotein-cholesterol (mmol/L)	1.009	0.392	6.631	0.010	2.742	1.272–5.911
Total protein (g/L)	−0.139	0.024	32.704	<0.001	0.870	0.830–0.913
Creatinine (μmol/L)	−0.043	0.009	20.387	<0.001	0.958	0.941–0.976
Total bilirubin (μmol/L)	−0.108	0.032	11.635	0.001	0.898	0.844–0.955
Constant	0.511	1.864	0.075	0.784	1.666	—

**Table 4:** Estimated performances of all single markers and combined markers by ROC curve.

	Sensitivity	Specificity	AUC	P	95% CI
Urea nitrogen	—	—	0.633	<0.001	0.583–0.683
Fasting blood-glucose	—	—	0.628	<0.001	0.578–0.678
Fructosamine	—	—	0.571	0.007	0.520–0.622
Total protein	—	—	0.618	<0.001	0.568–0.668
Creatinine	—	—	0.693	<0.001	0.646–0.740
High density lipoprotein-cholesterol	—	—	0.567	0.011	0.516–0.618
Total bilirubin	—	—	0.605	<0.001	0.555–0.655
Combined markers	0.806	0.636	0.810	<0.001	0.768–0.846
